# Novel *FSIP2* Variants Induce Super-Length Mitochondrial Sheath and Asthenoteratozoospermia in Humans

**DOI:** 10.7150/ijbs.76051

**Published:** 2023-01-01

**Authors:** Mingrong Lv, Dongdong Tang, Hui Yu, Hao Geng, Yiling Zhou, Zhongmei Shao, Kuokuo Li, Yang Gao, Senchao Guo, Chuan Xu, Qing Tan, Chunyu Liu, Rui Guo, Huan Wu, Zongliu Duan, Jingjing Zhang, Guanxiong Wang, Rong Hua, Feifei Fu, Kai Wang, Yuping Xu, Ping Zhou, Zhaolian Wei, Feng Zhang, Yunxia Cao, Xiaojin He

**Affiliations:** 1 Reproductive Medicine Center, Department of Obstetrics and Gynecology, the First Affiliated Hospital of Anhui Medical University, Hefei 230022, China.; 2 NHC Key Laboratory of Study on Abnormal Gametes and Reproductive Tract (Anhui Medical University), Hefei 230032, China.; 3 Key Laboratory of Population Health Across Life Cycle (Anhui Medical University), Ministry of Education of the People's Republic of China, Hefei 230032, China.; 4 Anhui Province Key Laboratory of Reproductive Health and Genetics, Hefei 230032, China.; 5 Biopreservation and Artificial Organs, Anhui Provincial Engineering Research Center, Anhui Medical University, Hefei 230032, China.; 6 Department of Obstetrics and Gynecology, Fuyang Hospital of Anhui Medical University, Fuyang 236112, China.; 7 Obstetrics and Gynecology Hospital, Institute of Reproduction and Development, Fudan University, Shanghai 200011, China.; 8 Shanghai Key Laboratory of Female Reproductive Endocrine Related Diseases, Shanghai 200011, China.; 9 Anhui Provincial Human Sperm Bank, the First Affiliated Hospital of Anhui Medical University, Hefei 230022, China.

**Keywords:** Infertility, asthenoteratozoospermia, mitochondrial sheath, *FSIP2*, ICSI

## Abstract

Asthenoteratozoospermia is one of the major factors for male infertility, whereas the causes of large numbers of cases are still unknown. We identified compound heterozygous variants of *FSIP2* in three unrelated individuals from a cohort of 105 patients with asthenoteratozoospermia by exome sequencing. Deleterious *FSIP2* variations caused severe disassembly of the fibrous sheath and axonemal defects. Intriguingly, spermatozoa in our study manifested “super-length” mitochondrial sheaths, increased levels of the mitochondrial sheath outer membrane protein TOMM20 and decreased mitochondrial ATP consumption. Dislocation or deletion of the annulus and reduction or dislocation of the annulus protein SEPT4 were also observed. While the lengthened mitochondrial sheaths were not presented in men harboring *SEPT4* variants. Furthermore, female partners of two of three men achieved successful pregnancies following intracytoplasmic sperm injection (ICSI). Overall, we presume that FSIP2 may not only serve as a structural protein of the fibrous sheath but also as an intra-flagellar transporter involving in the axonemal assembly, mitochondrial selection and the termination of mitochondrial sheath extension during spermatogenesis, and ICSI is an effective treatment for individuals with *FSIP2*-associated asthenoteratozoospermia.

## Introduction

Approximately one-sixth of couples of reproductive age suffer from infertility 1-3. According to surveys, about 50% of these cases are induced by male factors, defined as male infertility. Sperm defects, including oligozoospermia, asthenozoospermia, and teratozoospermia, are the most common causes of male infertility 4, 5. Asthenoteratozoospermia, a most common form of male infertility, is characterized by decreased sperm motility and abnormal sperm morphology and accounts for around 19% of male infertility cases 6, 7.

Teratozoospermia is characterized by the presence of morphologically abnormal sperm with head, neck, and/or flagella defects. Subsequently, morphological deformities cause reduced motility or absent sperm motility. The abnormal sperm flagella presents with absent, short, bent, coiled, or irregular flagella 8, 9. Human sperm flagellum contain three parts based on the unique accessory structures surrounding the axoneme: mid-piece, principal piece, and end-piece 9. The axoneme, an evolutionarily conserved motile cilia and sperm flagellum structure, comprises nine peripheral double-microtubules (DMT) enveloping two central pairs (CP), termed the “9+2” structure, throughout the flagellum 10. In the mid-piece, the axoneme is wrapped by outer dense fibers (ODFs) and a mitochondrial sheath (MS) that is replaced with the fibrous sheath (FS) in the principal piece, with no peri-axonemal structure in the end piece 9, 11. Defects in the axoneme, peri-axoneme, protein transport, or centriole assembly can lead to defective sperm flagella, resulting in asthenoteratozoospermia. For example, variations in several dynein axonemal heavy chain (DNAH) family member genes 12-17 and cilia and flagella associated protein (CFAP) genes can cause various structural abnormalities in the axoneme 18-21. Moreover, variations in *TTC21A* (MIM: 618429), *TTC29* (MIM: 618735), and *CFAP69* (MIM: 617949) can severely disrupt flagellar axoneme assembly through the intra-flagellar transport (IFT) and intra-manchette transport (IMT) pathways during spermiogenesis 22-24. In addition, bi-allelic variations in *DZIP1* (MIM: 608671) and *CEP135* can cause severe defects due to abnormal centriole assembly. Owing to high phenotypic variability, the genetic causes of asthenoteratozoospermia remain largely unknown 25, 26.

The fibrous sheath (FS) is the first accessory structure formed after axoneme initiation during sperm tail development. In mature sperms, the FS is composed of longitudinal columns connected by transverse ribs 27. The A-kinase anchoring proteins (AKAPs), namely, AKAP3, AKAP4, and AKAP14, are the major components of the FS. Variations in *AKAP3* and *AKAP4* cause dysplasia of the fibrous sheath (DFS) 28, 29. Fibrous sheath-interacting protein 2 (FSIP2) localizes to the FS and directly interacts with AKAP4 30. Variations in *FSIP2* lead to loss of components of central microtubules, including CP, and complete defects in the axoneme, resulting in the multiple morphological abnormalities of the sperm flagella (MMAF) phenotype presenting with absent, short, coiled flagella 31-34. Although more than 15 proteins have been found to be involved in the FS 9, how they assemble this unique cytoskeletal structure remains unclear.

The mitochondrial sheath (MS) is arranged helically over nine ODFs and an axoneme in the mid-piece of the sperm flagellum. In humans, formation of the MS needs the redistribution of mitochondria from a broad cytoplasmic distribution to the sperm tail, with the last 72-80 elongated mitochondria forming a helix of approximately 10-12 gyres 35, with two mitochondria per gyrus. The number of gyres and the length of the MS are strictly regulated during late spermatogenesis 27, 36. In mice, previous studies found that the mitochondria were accumulated in the midpiece regions of* Cfap157* null sperm 37. ARMC12 interacted with mitochondrial proteins VDAC2, VDAC3, and MIC60 as well as TBC1D21 and GK2, regulating the mitochondrial interlocking step 38. Similarly, deficiency of TBC1D21, a loose and inordinate mitochondrial sheath assembled a hairpin like sperm flagella, losing interacting with ACTB, TPM3, SPATA19, and VDAC3 39. In mitochondrial fission factor (*Mff*) mutant mice, mitochondrial sheaths of elongating spermatids and spermatozoa are disjointed, swollen mitochondria arranged loosely 40. *Glycerol kinase 2 (Gk2)*-deficiency sperm showed abnormal localization of crescent-like mitochondria 39, 41. *Fsip1*^-/-^ sperm revealed abnormal accumulation of mitochondria 42. In humans, abnormalities in spermatozoa, including shorter mid-pieces with fewer mitochondrial gyres, abnormal mitochondrial assembly, and structural defects in mitochondrial membranes, have been associated with asthenozoospermia 43-45. Moreover, except for specific flagellum defects present in the MMAF phenotype, variations in* CFAP65* (MIM: 614270),* CFAP58* (MIM: 619129), *EIF4G1* (MIM: 600495), and *DNHD1* (MIM: 617277) also cause sperm MS malformations 19, 46-48. However, the mechanisms underlying these defects during spermatogenesis are not clearly understood.

In this study, we used whole-exome sequencing (WES) to identify compound heterozygous variations in three unrelated subjects from a cohort of 105 individuals with asthenoteratozoospermia. *FSIP2* variations led to significant reductions in *FSIP2* mRNA and protein levels, resulting in the disruption or loss of FS. Subsequently, *FSIP2* deficiency was considered the leading cause of axoneme disorganization. Additionally, we found that *FSIP2* deficiency induced the elongation of the mitochondrial sheath and eliminated the annulus ring. The absence of annulus ring was not associated with the super-length MS in sperm from subjects harboring *SEPT4* variants. Furthermore, the partners of two patients achieved successful pregnancy outcomes following intracytoplasmic sperm injection (ICSI). Thus, we speculate that FSIP2 may serve as both a structural protein of the fibrous sheath and a component of intra-flagellar transporter complex participating in the assembly of flagellum and MS during spermatogenesis. Lastly, our study also provided clinical evidence that ICSI is an effective treatment for individuals with *FSIP2*-associated asthenoteratozoospermia.

## Materials and Methods

### Study participants

We enrolled 105 infertile men with asthenoteratozoospermia from the reproductive medicine center of the First Affiliated Hospital of Anhui Medical University. Detailed clinical manifestations of primary ciliary dyskinesia-related symptoms (i.e. sinusitis, pneumonia, and bronchitis) were reviewed and excluded. Clinical examination revealed normal male testicle size, external genitalia, hormone levels, and secondary sexual characteristics. Each individual had the chromosomal karyotype 46, XY. No Y chromosome microdeletions were identified. The present study was approved by the Ethics Committee of Anhui Medical University. All participants involved in this study signed the informed consents before collecting peripheral blood or semen samples.

### Whole-exome sequencing (WES)

Peripheral whole blood samples were collected from the asthenoteratozoospermia -affected individuals, and genomic DNA extraction and exome enrichment were performed. Briefly, qualified genomic DNA samples were sequenced on the Illumina HiSeq X-TEN platform. Raw data generated from each sample were aligned against the human reference genome (UCSC Genome Browser hg19) using the Burrows-Wheeler Aligner (BWA) algorithm after removing low-quality reads. All genomic variations, including single nucleotide variations and InDels, were annotated and filtered. Variant screening was conducted as previously described 49. ANNOVAR software ((Version: 2020-06-08) was used for functional annotation using information from various databases and bioinformatics tools, including OMIM, Gene Ontology, SIFT, PolyPhen-2, 1000 Genomes Project, and gnomAD 50. Pathogenic variants in genes, testis-specific expressed or biased expression in testis, were selected as candidates. In humans, testis-specific expression or biased expression in testis was defined as an average expression value of ≥ 5 reads per kilobase per million map reads in the testis and more than 2-folds of the average expression value in other tissues based on the GTEx. Candidate variants were validated using Sanger sequencing. The primers used to validate *FSIP2* variants are listed in Table S1.

### Sperm motility, morphology and viability measurements

Parameters of semen from healthy and infertile men were analyzed using computer-assisted sperm analysis based on WHO guidelines (6^th^ Edition) during routine biological examination 51. Briefly, semen samples were collected through masturbation after 3-7 days of sexual abstinence and examined after liquefaction at 37 °C for 30 min. Each individual's semen parameters were measured at least three times. H&E and Papanicolaou staining was used to analyze sperm morphology. Briefly, sperm samples were fixed with 4% paraformaldehyde, dehydrated, and then stained with H&E and Papanicolaou. At least 200 spermatozoa from each sample were assessed, based on WHO guidelines, to determine the percentage of morphologically abnormal spermatozoa. Each flagellum was classified into a morphological category based on the main flagellar abnormality.

Regarding to the sperm vitality test, we used eosin staining partly based on 6th WHO guidelines. Briefly, mix a 50 μl aliquot of semen sample with an equal volume of eosin-suspension in a test tube, and wait for 30 seconds. Make a smear on a glass slide and examine the slide with bright field optics at ×1000 magnification and oil immersion. Evaluate at least 200 spermatozoa, to achieve an acceptably low sampling error. Spermatozoa with red or dark pink heads are considered dead, whereas spermatozoa with white heads are considered alive.

### Scanning electron microscopy (SEM) and transmission electron microscopy (TEM) analyses

Fresh semen samples from normal controls and men carrying *FSIP2* variants were washed three times with 1× phosphate-buffered saline (PBS), centrifuged at 2500 rpm for 10 min and then fixed with 2.5% glutaraldehyde (pH 6.9) for 2 h at 4 °C.

For SEM, fixed spermatozoa were progressively dehydrated using increasing concentrations of ethanol (30%, 50%, 70%, 80%, 90%, and 100%) and then dried in a K850 CO_2_ critical point dryer (Quorum Technologies, Lewes, UK). The specimens were then coated with metal particles using a Cressington 108 Auto carbon coater (Cressington Scientific Instruments Ltd, Watford, UK) and analyzed under a GeminiSEM 300 scanning electron microscope (ZEISS, Oberkochen, Germany).

For TEM, fixed spermatozoa were post-fixed with 1% osmium tetroxide for 2 h at 4 °C, dyed with 2% uranium acetate for 2 h, and dehydrated in a graded series of ethanol (50%, 70%, 90%, and 100%) and 100% acetone. The fixed spermatozoa were then embedded in EPON 812 epoxy resin. Finally, the embedded spermatozoa were sliced into ultrathin sections (100nm thick), stained with lead citrate, and observed under a Talos L120C G2 transmission electron microscope (Thermo Fisher Scientific, Waltham, MA, USA).

### Real time PCR analysis

Sperm samples were collected from three fertile men and three patients carrying *FSIP*2 variants. At least three semen samples from each patient were collected over 3 months through masturbation after 3-7 days of sexual abstinence. Total RNA was extracted from the sperm samples using TRIzol reagent (Invitrogen, Waltham, MA, USA). Approximately 1 μg of the obtained RNA was converted into cDNA using SuperScript III Reverse Transcriptase and oligo (dT) primers (TaKaRa Bio, Kusatsu, Shiga, Japan). The cDNA was used as a template in subsequent PCR amplification with transcript-specific primers (Table S1). Quantitative real-time polymerase chain reaction (RT-qPCR) was performed, with β-actin as an internal control. Data were analyzed using the 2^-ΔΔCt^ method.

### Immunofluorescence analysis

Immunofluorescence (IF) analysis was performed using sperm cells from healthy controls and patients carrying *FSIP2* variants. The procedures, including sample fixation, smear preparation, antigen blocking, and antibody incubation, were performed as previously described 25. The following were used as primary antibodies: rabbit polyclonal anti-SEPT4 (Affinity, DF13393, 1:200), rabbit polyclonal anti-TOMM20 (Proteintech, 11802-1-AP, 1:400), rabbit polyclonal anti-AKAP4 (Sigma-Aldrich, HPA020046, 1:200), rabbit polyclonal anti-SPAG6 (Sigma-Aldrich, HPA038440, 1:200), rabbit polyclonal anti-FSIP2 (Sigma-Aldrich, HPA036139, 1:50), mouse polyclonal anti-TOMM20 (Proteintech, 66777-1-Ig,1:200), rabbit polyclonal anti-DNAI2 (Proteintech, 17533-1-AP, 1:200), rabbit polyclonal anti-IFT88 (Proteintech, 13967-1-AP, 1:200), rabbit-polyclonal anti-IFT74 (Sigma Aldrich, HPA020247, 1:200), rabbit-polyclonal anti-IFT20 (Abcepta, Cat# AP5133C, 1:200), and mouse monoclonal anti-acetylated-tubulin (Sigma-Aldrich, T6793, 1:200). After washing with PBS, slides were incubated for 1 h at 37 °C with highly cross-adsorbed secondary antibodies: Alexa Fluor 488 anti-mouse IgG (Yeasen Biotechnology, 34106ES60, 1:500) and Alexa Fluor 594 anti-rabbit IgG antibodies (Jackson ImmunoResearch Inc., 111-585-003, 1:500). The nuclei were then labeled with Hoechst nuclear stain (Thermo Fisher Scientific Inc., 62,249, 1:1000) and incubated at 37 °C for 5 min. Images were captured using the LSM 800 confocal microscope (Carl Zeiss AG).

### Western blot (WB) analysis

Proteins were isolated from spermatozoa samples using RIPA lysis buffer (Beyotime Biotechnology) and used for western blot analysis. The lysates were separated on a 10% polyacrylamide gel using sodium dodecyl sulfate-polyacrylamide gel electrophoresis and transferred onto polyvinylidene fluoride membranes. To block non-specific binding to the membranes, the blots were blocked with 5% non-fat milk in Tris-buffered saline containing 0.1% Tween-20 for 1 h at 25 °C. The blots were then incubated with shaking overnight at 4 °C with primary antibodies diluted in TBST. Rabbit polyclonal SEPT4 (1:1000), rabbit polyclonal anti-TOMM20 (1:1000), rabbit polyclonal anti-AKAP4 (1:1000), rabbit polyclonal anti-SPAG6 (1:1000), rabbit polyclonal anti-DNAI2 (1: 1000), rabbit polyclonal anti-IFT88 (1:1000), rabbit polyclonal anti-IFT74 (1:1000), rabbit polyclonal anti-IFT20 (1:1000), and mouse polyclonal anti-β-actin were used. The blots were subsequently washed in TBST and incubated at 25 °C for 1 h with 1:5000 dilutions of HRP-conjugated secondary antibody. Enhanced chemiluminescence (Biosharp, BL520A) was used to visualize target proteins, and β-actin was used as the loading control.

### Measurement of ATP content

The amount of spermatozoa ATP was examined using CellTiter-Glo luminescent cell viability assay Kit (Promega, USA) according to the manufacturer's instructions. After 30 min incubation to allow for sperm liquefaction (at 37 °C in 5% CO_2_ atmosphere), we immediately measured ATP contents (time point 0) in SpectraMax iD3 (Molecular Devices, US). A second measure was similarly performed after sperm incubation for another 1 h. All measures were performed in triplicated for each semen sample. Three semen samples were collected three times from each patient. ATP content was calculated using an ATP standard curve (dilutions of ATP in medium from 10 μM to 10 nM).

### Assessment of sperm mitochondrial membrane potential

Mitochondrial membrane potential was assessed using JC-1 assay kit according to the instructions described previously 52. JC-1-stained sperm samples were analyzed by flow cytometry (BD FACSVerse™ Flow Cytometer (BD Biosciences, USA)) collecting 50000 cells. JC-1 exists inside the mitochondria in the J-aggregates form and emits red fluorescence (PI channel), representing high mitochondrial membrane potential (MMP). Whereas, it remains in the monomer form and emits green fluorescence (FITC channel), representing in a low MMP state.

### Statistical analysis

Data obtained from RT-qPCR were analyzed using GraphPad Prism (GraphPad Software, San Diego, CA, USA), and fold-change in RNA expression was calculated using the 2^-ΔΔCt^ method. Morphological data from two participants were evaluated and then statistically rated, with statistical significance set at p < 0.05.

## Results

### Identification of deleterious *FSIP2* variants in three individuals with asthenoteratozoospermia

**Figure 1:**
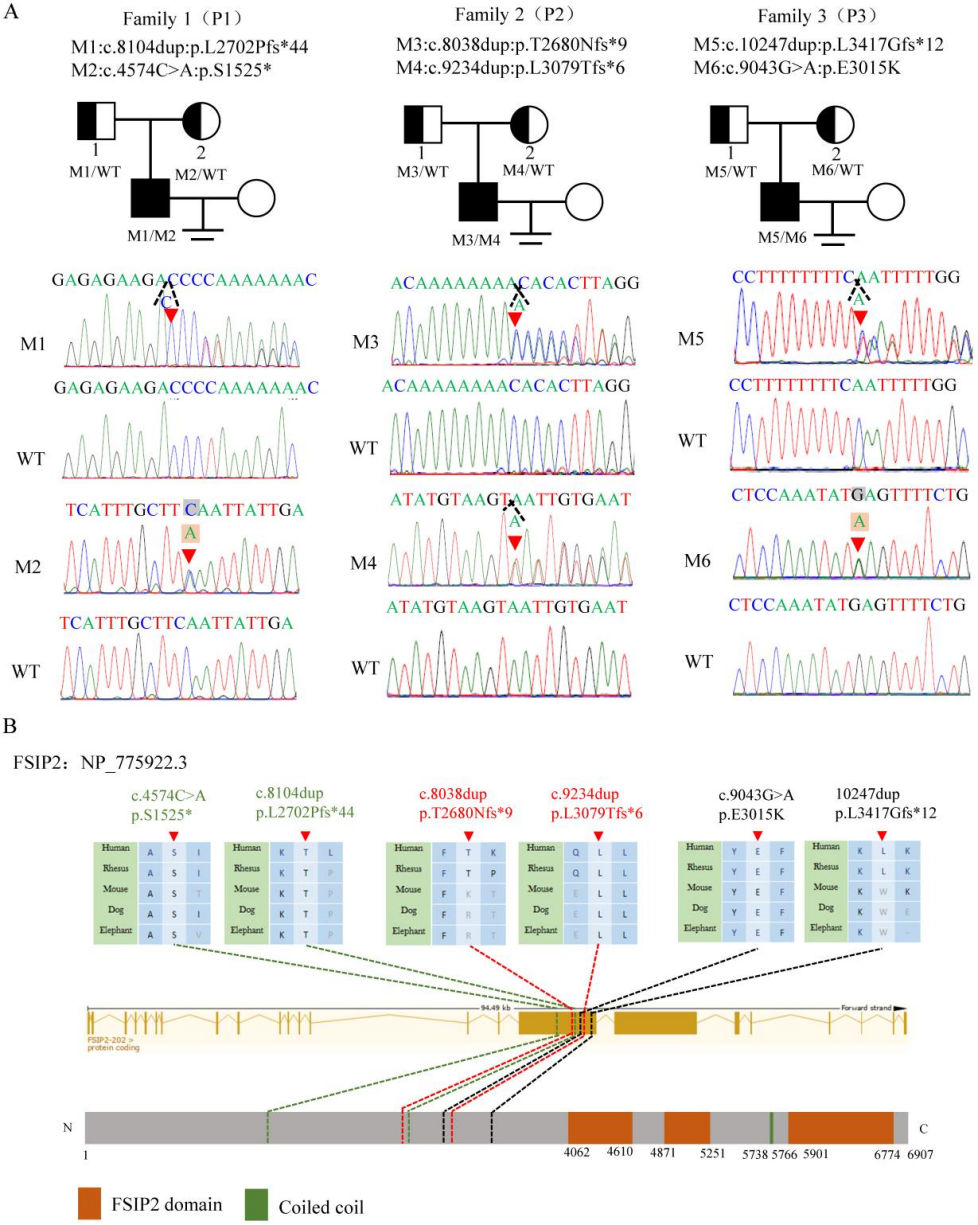
** Compound heterozygous variants of *FSIP2* in three families with MMAF-affected asthenoteratozoospermia. (A)** Novel *FSIP2* variants (M-M6) were identified in three subjects with MMAF (P1, P2 and P3). The heterozygous parental carriers of all *FSIP2* variants are shown. Arrows (red) show the positions of novel *FSIP2* variants identified in the present study. The triangle dotted lines (black) show positions of duplicated bases and squares stand for the bases (grey) were replaced by another ones (light orange). **(B)** Schematic representation of exons and protein product of *FSIP2*gene. Arrows (red) and dotted lines show (green, red and black) the positions of novel *FSIP2* variants identified in the present study. Sequence alignments show conservation of the affected amino acid across different species. Orange squares stand for the FSIP2 domains and green square stands for the typical protein-folding motif called coiled-coil (CC) domain according to the NCBI browser. WT, wild type, M, mutation, P, patient.

In this study, WES and bioinformatics analyses were performed according to a previously described protocol [19]and three compound heterozygous *FSIP2* variants (NM_173651.4) were identified in three unrelated subjects. All identified variants were verified using Sanger sequencing. Compound heterozygous c.8104dup (p.L2702Pfs*44) and c.4574C>A (p.S1525*) in family 1 (P1), compound heterozygous c.8038dup (p. T2680Nfs*9) and c.9234dup (p. L3079Tfs*6) in family 2 (P2), and compound heterozygous c.10247dup (p. 3417Gfs*12) and c.9043G>A (p.E3015K) in family 3 (P3) were inherited from heterozygous parents, consistent with an autosomal recessive mode of inheritance (Fig. 1A). FSIP2 protein is one of the main component of the flagella FS. It contains three FSIP2 domains and a coiled-coil domain. The frameshift variants (M1-M5) identified in this study presumably result in premature termination codons and truncated proteins, suggesting their strong deleterious effects (Fig. 1B, Table 1). The remaining missense variant was highly conserved across species and predicted to be damaging (Fig. 1B, Table 1). Notably, analysis of the prevalence of these variants in the 1000 Genomes and gnomAD databases revealed that the variants were either extremely rare or absent in the general population (Table 1). Therefore, we hypothesized that *FSIP2* variants identified here may be responsible for the observed infertility phenotypes.

### *FSIP2* variants are associated with the sperm super-length mitochondrial sheaths and thicker mid-pieces

**Figure 2:**
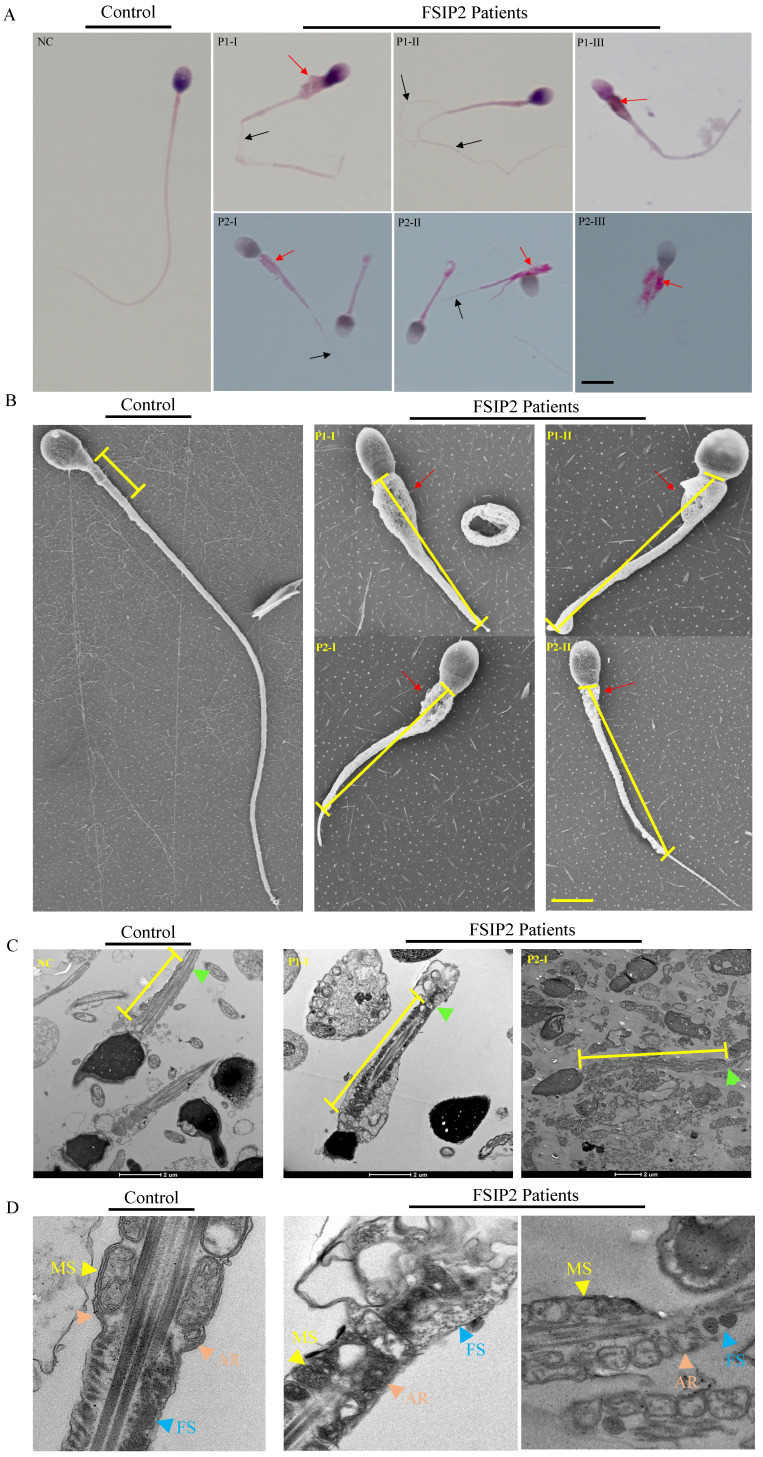
** Morphology and ultrastructure analysis of spermatozoa in the fertile males and men harboring *FSIP2* variants. (A)** Morphological sperm defects analyzed through light microscopy (100×, oil objective, scale bar: 10μm) by H&E (NC and P1) and Papanicolaou (P2) staining. Spermatozoa from the fertile control showed normal long and smooth flagella, whereas abnormal thick neck (red arrow), multiple thin flagella (black arrow), short, coiled flagella, flagella of angulation and irregular caliber could be obviously observed in the spermatozoa of *FSIP2*-deficient men. **(B)** SEM analysis of sperm morphology from a normal control and *FSIP2*-deficient subjects. Most spermatozoa from men harboring *FSIP2* variants presented thickened neck (red arrow) and lengthened mid-piece (yellow line). Scale bar: 5μm. **(C and D)** Electron micrographs of longitudinal sections of sperm flagellar mid-piece of a control individual and men harboring *FSIP2* variants. The normal sperm mid-piece had a regularly arranged MS (yellow arrows) with the length of about 5μm adjacent to the FS connected by annulus ring (brown arrows). While, the extension of mitochondrial sheath in* FSIP2*-deficient subjects showed a significantly lengthened with destroyed or absent fibrous sheath (blue arrows). MS, mitochondrial sheath, FS, fibrous sheath, P1, patient 1, P2, patient 2, Scale bars: 2μm and 1μm.

Sperm parameters were examined in the clinical laboratories during routine biological examinations of patients, in accordance with the 6^th^ World Health Organization (WHO) guidelines 51. Normal semen volumes and sperm concentrations were observed in patients carrying *FSIP2* variants; however, sperm motility and progressive motility were lower (Table 2). The morphology of sperm cells from patients carrying *FSIP2* variants was assessed using H&E and Papanicolaou staining (Fig. 2A), and morphologically abnormal spermatozoa were evaluated according to WHO guidelines. Sperm morphological analysis showed that most of spermatozoa displayed with elongated mid-pieces connected to the short or thin principal pieces with or without dysplastic fibrous sheath surrounding (Fig. 2A and Table 2). Approximately, one third of sperm necks were thickened by encapsulated in excess residual cytoplasm (ERC) (Table 2). Meanwhile, the defective sperm head mainly presented with amorphous head and vacuolated acrosome (Table 2). SEM confirmed the phenotypes observed above in two cases. Intriguingly, SEM revealed thicker necks and longer mitochondrial sheaths in the mid-pieces and dysplastic sperm FS in the principal pieces in individuals carrying *FSIP2* variants compared with normal controls (Fig. 2B). We also investigated the sperm flagellar ultrastructure in individuals carrying* FSIP2* variants using TEM. Longitudinal sections of sperm flagella observed using TEM revealed that, compared with normal controls, the MS was more than twice as long in individuals carrying *FSIP2* variants. Additionally, compared with the regularly arranged mitochondrial sheath and the fibrous sheath connected by the annulus observed in normal sperm, sperm in individuals carrying* FSIP2* variants had no annulus and exhibited severe FS dysplasia (Fig. 2C and 2D). These findings suggest that the *FSIP2* variants not only affect the assembly of the FS but also affect the elongation of the mitochondrial sheath.

### Severe disorganization of axonemal and fibrous sheath structures in individuals carrying *FSIP2* variants

**Figure 3:**
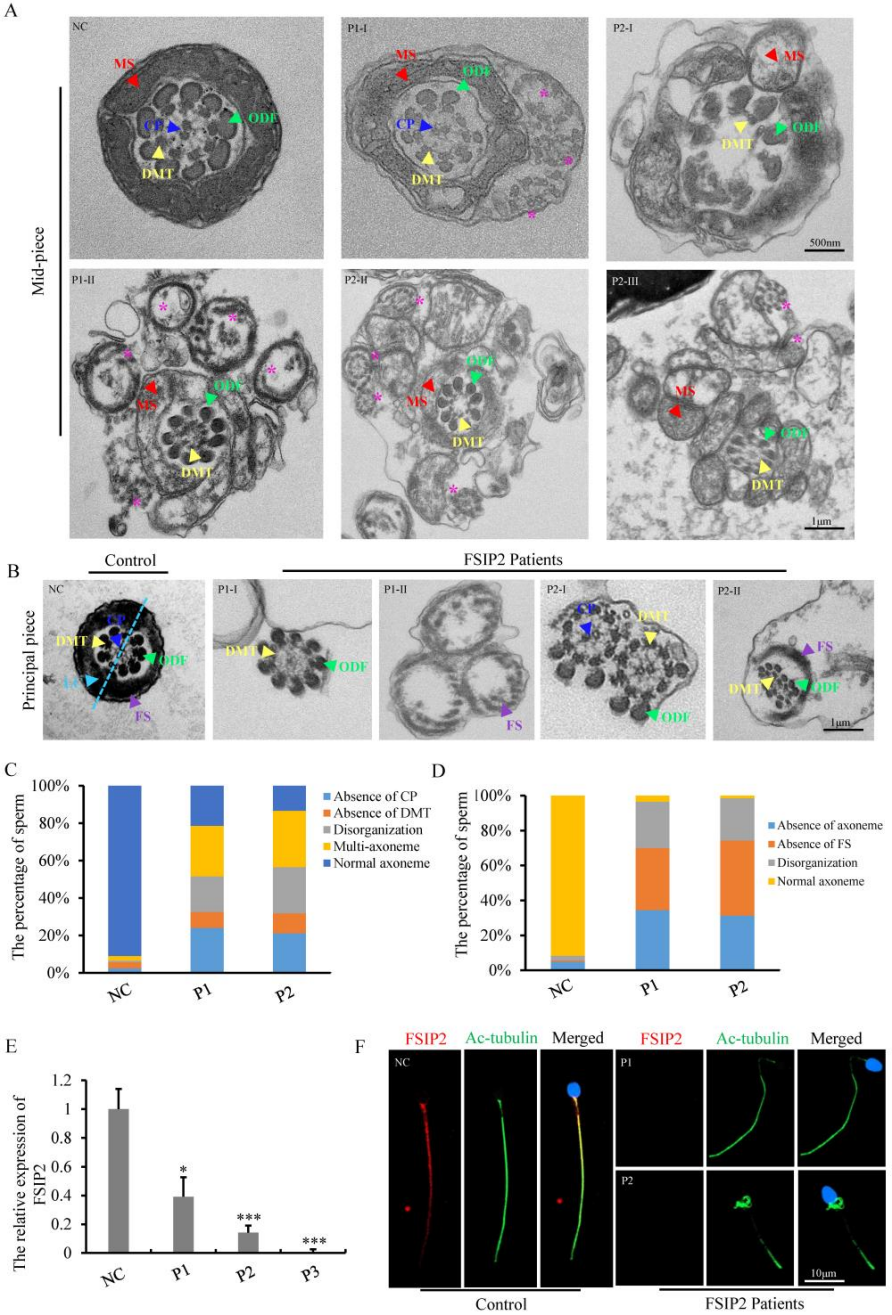
***FSIP2* deficiency associated with sperm axoneme malformations and central pair absence. (A)** Transmission electron microscopy (TEM) of analysis of sperm mid-piece ultra-structure in *FSIP2*-deficient human subjects. Cross section of sperm from a normal subject presented the typical normal “9+2” microtubule structure with CP (blue arrow), DMT (yellow arrow), MS (red arrow) and arrangement of ODF (green arrow). Compared with the control subject, mid-piece with absence of CP, or surrounded with multiple axoneme (pink stars) without FS or/and empty FS or increased mitochondria can be observed in the subjects with *FSIP2* variants, CP, central pair, DMT, doublets of microtubules, MS, mitochondrial sheath, ODF, outer dense fibers, Scale bars: 1μm. **(B)** TEM of analysis of sperm principal piece ultra-structure in *FSIP2*-deficient subjects. In the control subject, the axoneme is surrounded by seven ODFs and by the FS composed of two longitudinal columns (LC, marked by light blue dotted line and arrow) connected by circumferential ribs. Cross-sections of sperm flagellum from* FSIP2* deficient subjects, the FS is absent (P1-I and P2-I), dysplastic without LC (P2-II) or dramatically disorganized (P1-II and P2-II). Various axonemal anomalies can be observed including the lack of axoneme or CP, or complete axonemal disorganization. Additionally, alterations of ODFs is also seen with missing or fully disorganized ODFs. Scale bars: 1μm. **(C and D)** Quantification of different categories of flagellar mid-piece and principal piece ultrastructural defects. Total cross-section numbers for quantification in the control subject and men harboring *FSIP2* variants (P1 and P2) were 121, 178 and 113, respectively. Cross-sectional defects were classified into four categories: absence of CP (24.2% & 21.2% for subjects P1 and P2), absence of DMT (8.4% & 10.6%), disorganization (19.1% & 24.8%) and multi-axoneme (27% & 30.1%). Total cross-section numbers for quantification in the control subject and men harboring *FSIP2* variants (P1 and P2) were 120, 87 and 70, respectively. Cross-sectional defects were classified into three categories: absence of axoneme (34.5% & 31.4%), absence of FS (35.6% &41.9%) and disorganization (26.4% &24.3%). **(E)** The relative mRNA expression levels of* FSIP2* in sperm from *FSIP2*-deficient subjects and three control subjects. The *FSIP2* mRNA level of *FSIP2*-deficient subjects was significantly reduced compared with that in the controls. ^*^ P<0.05, ^***^ P < 0.001. **(F)** FSIP2 immunofluorescence assays of human sperm. Immunofluorescence staining reveals the absence of specific staining of FSIP2 in sperm flagella from patients with *FSIP2* compound heterozygous variants. Anti-ac-tubulin (green) marked the sperm flagella. The nuclei of spermatozoa were Hoechst-labeled (blue). Scale bars: 10μm.

TEM was used to assess the effect of *FSIP2* compound heterozygous variations on the sperm flagellar ultrastructure. Compared with fertile controls, a variety of ultrastructural defects were observed in the mid-piece of spermatozoa from patients with *FSIP2* deficiency. We found integrated and regularly arranged DMT and ODF in patients. The CP was missing from the mid-pieces of 24% of flagella from P1 and 21% of flagella from P2. In addition, multiple FS with disassembled DMTs and ODFs, multiple bare axonemes, or some doublets of microtubules (DMT) scattered throughout excess residual cytoplasm were also observed surrounding the mid-piece, presenting with thick neck, which accounted for about 27% in P1 and 30% in P2. The remaining cross-sections had highly disorganized mid-pieces (19% and 25%) (Fig. 3A and 3C). As shown in Fig. 3B, in the normal principal piece, the axoneme is surrounded by seven ODFs and flanked by an FS composed of two longitudinal columns connected by circumferential ribs (ODFs 3 and 8 are replaced by two longitudinal columns). Analysis of individuals with *FSIP2* deficiency showed that 36% and 43% of the cross-sections had disordered FS surrounding the axoneme; 34% and 31% of the analyzed cross-sections lacked axonemes and regularly arranged ODFs, while others exhibited disorganized axonemes and ODFs. Additionally, nine ODFs were observed around the axoneme in the cross-sections of some principal pieces, suggesting that the 3^rd^ and 8^th^ ODF were not replaced by longitudinal columns (Fig. 3B and 3D).

*FSIP2* is exclusively expressed in human and mouse testis 31. To understand the pathogenicity associated with *FSIP2* variants, we examined the expression and localization of FSIP2 in sperm cells collected from normal controls and patients carrying *FSIP2* variants using RT-qPCR and immunofluorescence. RT-qPCR results showed that *FSIP2* mRNA expression was significantly lower in patients carrying *FSIP2* variants than in normal controls (Fig. 3E). As shown in Fig. 3F, the FSIP2 staining signal was strong in the flagella of control sperm cells from fertile individuals, but the corresponding signal was almost absent in spermatozoa of individuals carrying *FSIP2* variants The FSIP2 antibody epitope is located after the mutation for P1 and P2. A possible truncated protein is therefore not recognized by the antibody, or possible due to nonsense-mediated mRNA decay triggered by premature translation termination. Collectively, these variations had a negative effect on FSIP2 expression, potentially disrupting sperm flagellar development and leading to the asthenoteratozoospermia phenotype.

### *FSIP2* deficiency is associated with increased mitochondrial protein, decreased ATP consumption and altered distribution of annulus components

**Figure 4:**
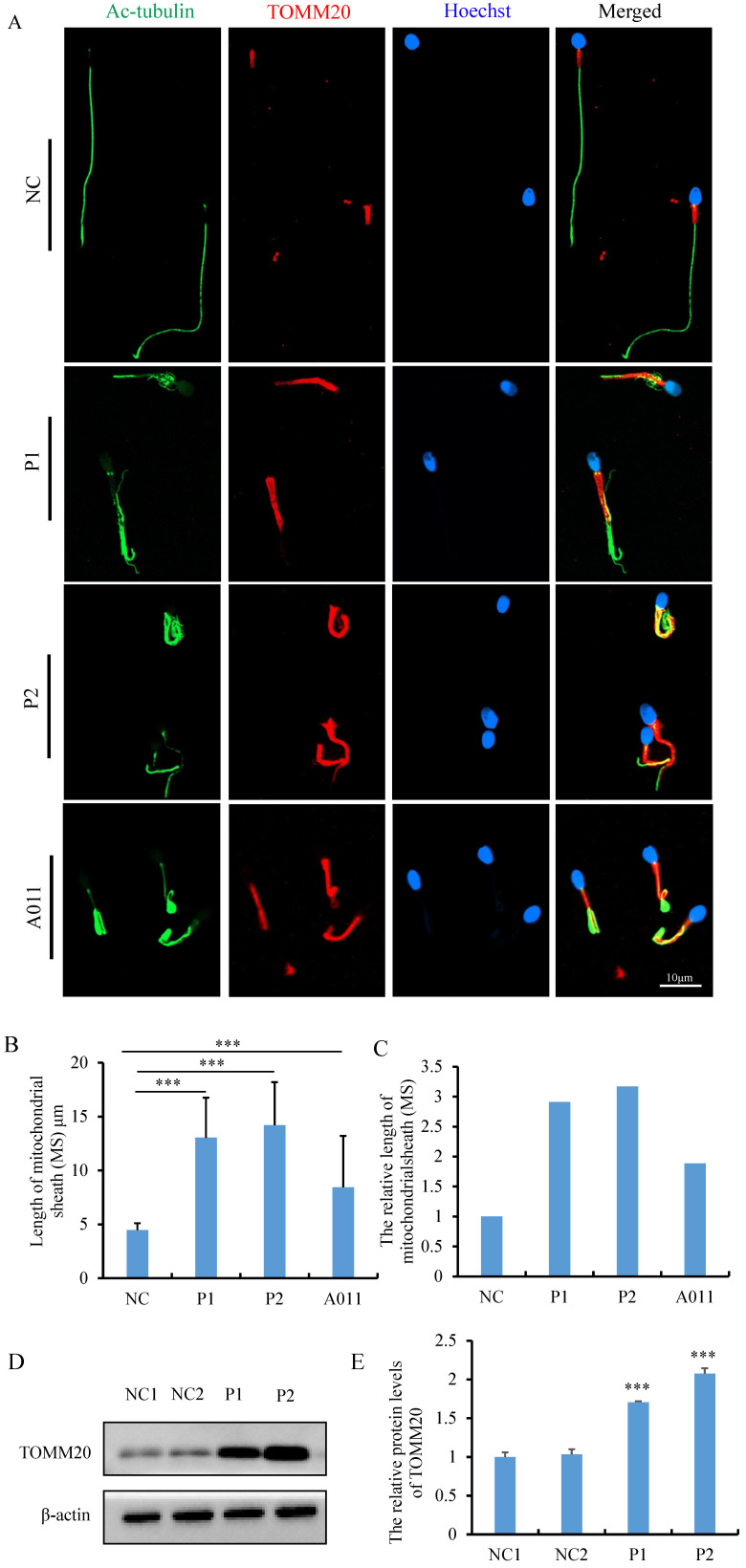
The measurement and analysis of MS length and levels of TOMM20 in sperm from controls and* FSIP2* patients. **(A)** The immunofluorescence staining analysis of TOMM20 in control subjects and *FSIP2* patients. Anti-TOMM20 (red) marked the sperm MS. Anti-ac-tubulin (green) marked the sperm flagella. The nuclei of spermatozoa were Hoechst-labeled (blue). Scale bars: 10μm. **(B-C)** Quantification of the length of MS in sperm from controls and *FSIP2* patients according to the TOMM20 signals. The measurements were performed using the LSM 800 confocal microscope image processing and quantification tools. 115, 75, 111 and 113 sperm from normal control, P1, P2 and A011were used to estimate the length of the MS. The average length of mitochondrial sheath was 4.47±0.63μm in sperm from normal controls, while, which were 13.04±3.71μm (P1), 14.20±3.99μm (P2) and 8.43 ±4.77μm (A011) of sperm from subjects harboring* FSIP2* variants, 2-3 times of that in control sperm. **(D-E)** The levels of TOMM20 in sperm cells from normal individual and *FSIP2*-deficient subjects analyzed by western blot. The abundances of TOMM20 were increased nearly two times in men harboring *FSIP2* variants when compared with those of the control individual. β-actin was used as internal reference.

Morphological and ultrastructural analyses of spermatozoa from individuals carrying *FSIP2* variants indicated that FSIP2 may play an essential role in mitochondrial sheath assembly and sperm flagellar formation. To investigate the effect of *FSIP2* variations on the mitochondrial sheath assembly state, we evaluated expression levels and localization of the mitochondrial outer membrane protein TOMM20. In normal sperm, TOMM20 staining signals were concentrated outside the mid-piece, the length of which were about 5 μm, approximately the same length as the sperm head. Conversely, the corresponding staining signals showed that the length of mid-pieces were 13.04±3.71 μm in sperm from P1 and 14.20±3.99 μm in sperm from P2, 2-3 times of that in control sperm (Fig. 4A, B and C). To determine whether the lengthened mitochondrial sheath was associated with *FSIP2* variants, as opposed to being a hallmark of the MMAF phenotype, we also performed TOMM20 immunofluorescence assays on sperm cells from individuals with *FSIP2* variants (A011) reported previously 32 and subjects with MMAF who carried variants in *DNAH10* and *TTC21A* genes. The length of TOMM20 staining signal in sperm from A011 (8.43 ±4.77μm) was also increased significantly, about twice that of normal sperm (Fig. 4A, B and C). While, TOMM20 staining signals of sperm cells from individuals harboring *DNAH10* and *TTC21A* variants were comparable with those observed in fertile controls (Fig. S1). Consistently, WB staining showed that levels of TOMM20 protein were higher 1-2 times in P1 and P2 than those in normal controls (Fig. 4D and E). In addition, ac-tubulin immunofluorescence staining showed that axonemal malformations, including disruption of the DMT structure and the growth of multiple flagella from the neck, which then surrounded the mitochondria, were observed in many spermatozoa in individuals carrying *FSIP2* variants (Fig. 4A). Overall, these findings were in accordance with the SEM and TEM observations.

**Figure 5:**
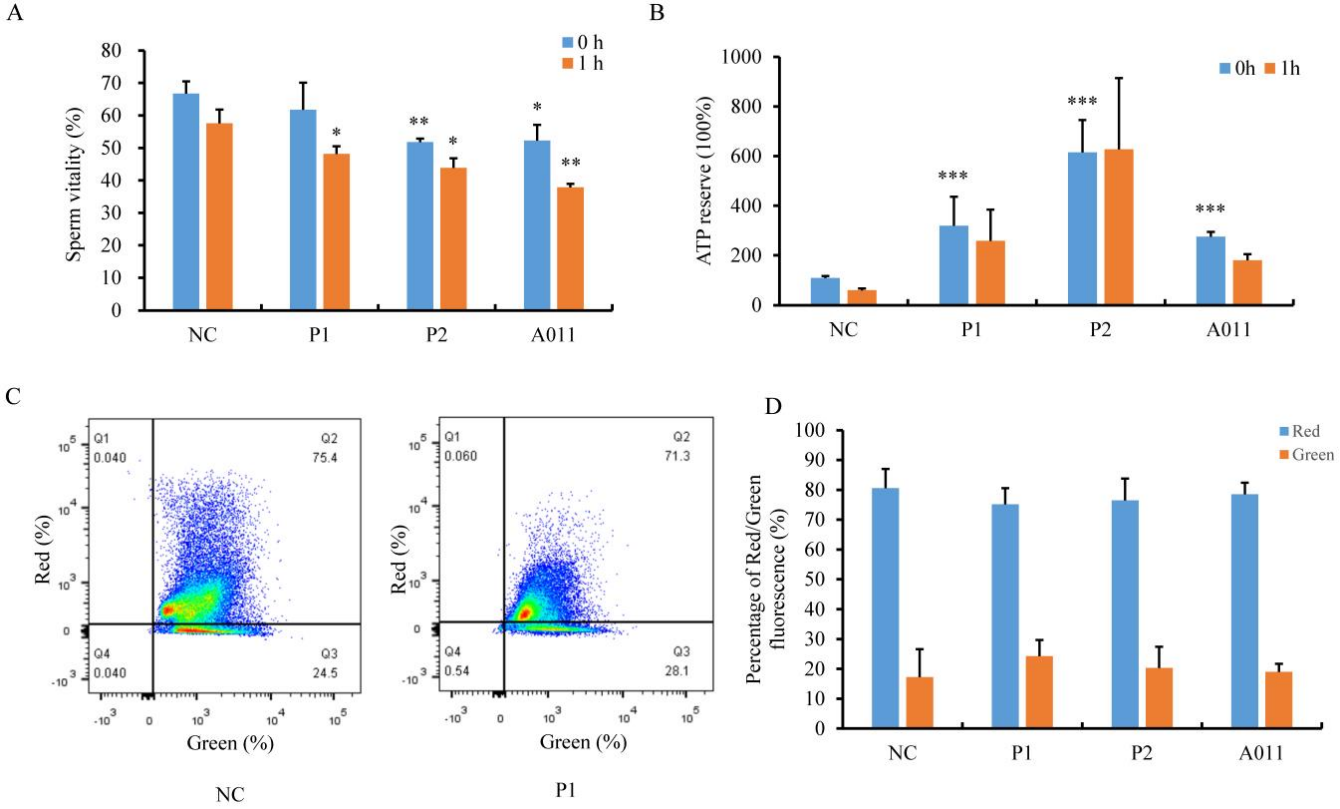
** The assessment of sperm mitochondrial function in normal individual and *FSIP2*-deficient subjects. (A)**The percentage of live spermatozoa was calculated in normal controls and patients. The liquefied spermatozoa were stained with eosin immediately (t=0) or 1 hour later (t=1), the total number and proportion of stained and unstained spermatozoa were calculated. Student t-test; n=3, n presents the number of repetitions,^ *^ P<0.05, ^**^ P < 0.01. **(B)** ATP reserve and consumption of sperm from control and patients. ATP content was measured by a luciferase method immediately after liquefaction (t=0) and after 1hour of incubation at 37℃ in 5% CO_2_ atmosphere (t=1). **(C and D)** Spermatozoa mitochondrial membrane potential (MMP) was examined by flow cytometry acquisition for JC-1-stained (a marker of MMP) and statistically analyzed. Red fluorescence presented the high MMP and green fluorescence indicated the low MMP.

Next, we attempted to determine the function of the lengthened MS. First, we examined sperm viability by staining liquefied spermatozoa with eosin immediately (t=0) or 1 hour later (t=1). We observed that percentages of live spermatozoa were decreased in P2 and A011, and more dead spermatozoa were found after 1h in individuals carrying *FSIP2* variants compared with normal controls (Fig. 5A). We then measured ATP content and ATP consumption in sperm from fertile controls and patients. Unexpectedly, spermatozoa from individuals with *FSIP2* variants contained far more ATP than normal controls. After incubation at 37 °C for 1h, the ATP levels of normal sperm was decreased by more than 50%, whereas, ATP consumptions were considerably lower in sperm from *FSIP2*-deficient patients (P1 and P2) than normal controls, and the ATP consumption was not affected severely in A011(Fig. 5B). Moreover, we also analyzed the mitochondrial membrane potential (MMP) through FACS using JC-1 staining of spermatozoa from controls and *FSIP2*-deficient patients. As shown in Fig. 5C and D, the MMPs were reduced slightly in sperm from *FSIP2*- deficient patients compared with control. These findings revealed that deficiency of *FSIP2* resulted in mitochondrial dysfunction in human sperm.

**Figure 6:**
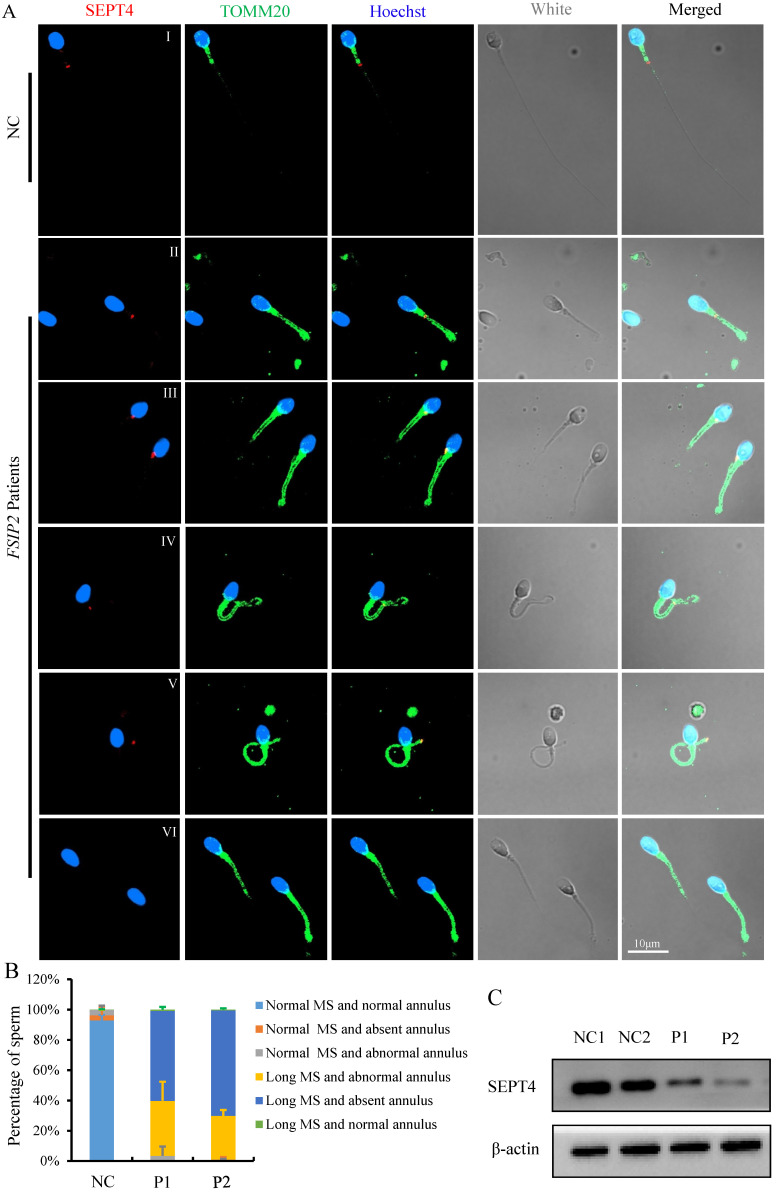
** The immunofluorescence staining analysis of the presence and localization of sperm annulus ring in normal individual and *FSIP2*-deficient subjects. (A)** The presence and localization of SEPT4 and TOMM20 staining signals in normal individual and *FSIP2*-deficient subjects. In normal sperm, the SEPT4 staining signal was located at the end of the MS, while the presence and localization of SEPT4 were disrupted in most of the analyzed sperm from *FSIP2* deficiency subjects, including presenting on the MS, proximal region of MS, distal region of MS and absence of SEPT4 on the sperm flagella, which accompanied by MS elongation. Anti-TOMM20 (green) marked the sperm MS. Anti-SEPT4 (red) marked sperm annulus ring. The nuclei of spermatozoa were Hoechst-labeled (blue). Scale bars: 10μm. **(B)** Quantification of different categories of sperm annulus ring and MS defects. Immunofluorescence (IF) analysis was performed using sperm cells from three fertile controls and patients carrying *FSIP2* variants (patient 1 (P1) and patient 2 (P2)). At least three semen samples from each patient were collected over 3 months through masturbation after 3-7 days of sexual abstinence. More than 200 spermatozoa from each sample were analyzed. The average sperm numbers for quantification in the control subjects and men harboring *FSIP2* variants (P1 and P2) were 240, 222 and 228, respectively. Sperm annulus ring and MS defects were classified into five categories: normal MS and absent annulus (0 & 0.8%), normal MS and abnormal annulus (0 & 0.2%), long MS and abnormal annulus (38.4% & 30.4%, III-V), long MS and absent annulus (60.7% & 69.2%, VI) and long MS and normal annulus (0.9% & 0.4%, II). **(C)** The levels of SEPT4 in sperm cells from normal individual and *FSIP2*-deficient subjects analyzed by western blot. The levels of SEPT4 were reduced in men harboring *FSIP2* variants. β-actin was used as internal reference.

The mitochondrial sheath in the sperm mid-piece is the final piece of sperm flagella that is formed during spermatogenesis. The length of mid-piece and the number of gyres are strictly regulated. The mechanisms that drive such processes during spermatogenesis have received little attention. To study the potential factors affecting spermatozoa MS length in individuals carrying the *FSIP2* variants, we used immunofluorescence to analyze the presence and localization of the annulus protein SEPT4, a critical component of the annulus ring that separates the middle and principal pieces. Using fluorescence microscopy, we detected SEPT4 as a dot located at the end of spermatozoa MS in healthy controls (Fig. 6A I). However, the presence and localization of SEPT4 signals were disrupted in most of sperms from *FSIP2* deficient individuals (P1 and P2) including presentation on the MS (0.9% & 0.4%, Fig. 6A II), the proximal region of the MS (0.7% & 0.6%, Fig. 6A III), and the distal region of the MS (37.7% & 29.8%, Fig. 6A IV-V and B). More than half of the analyzed sperms, SEPT4 signals were missing from the sperm flagella (60.7% & 69.2%, Fig. 6A VI and B), which were in accordance with the result that SEPT4 levels were lower in individuals with *FSIP2* deficiency (Fig. 6C). To determine whether the dislocation or absence of annulus ring was the direct cause of the lengthened MS, we detected the length of MS in men harboring *SEPT4* variants. As show in Fig. S2A, the SEPT4 staining signals were absent in men harboring *SEPT4* variants, while the lengthened MS were not observed (Fig. S2B). Therefore, our results indicated that the lengthening of spermatozoa MS in individuals with *FSIP2* deficiency may be due to dislocation or absence of the FS, not the annulus ring.

### Individuals carrying *FSIP2* variants lack AKAP4 protein and have altered distribution of axonemal components and IFT-B complexes

**Figure 7:**
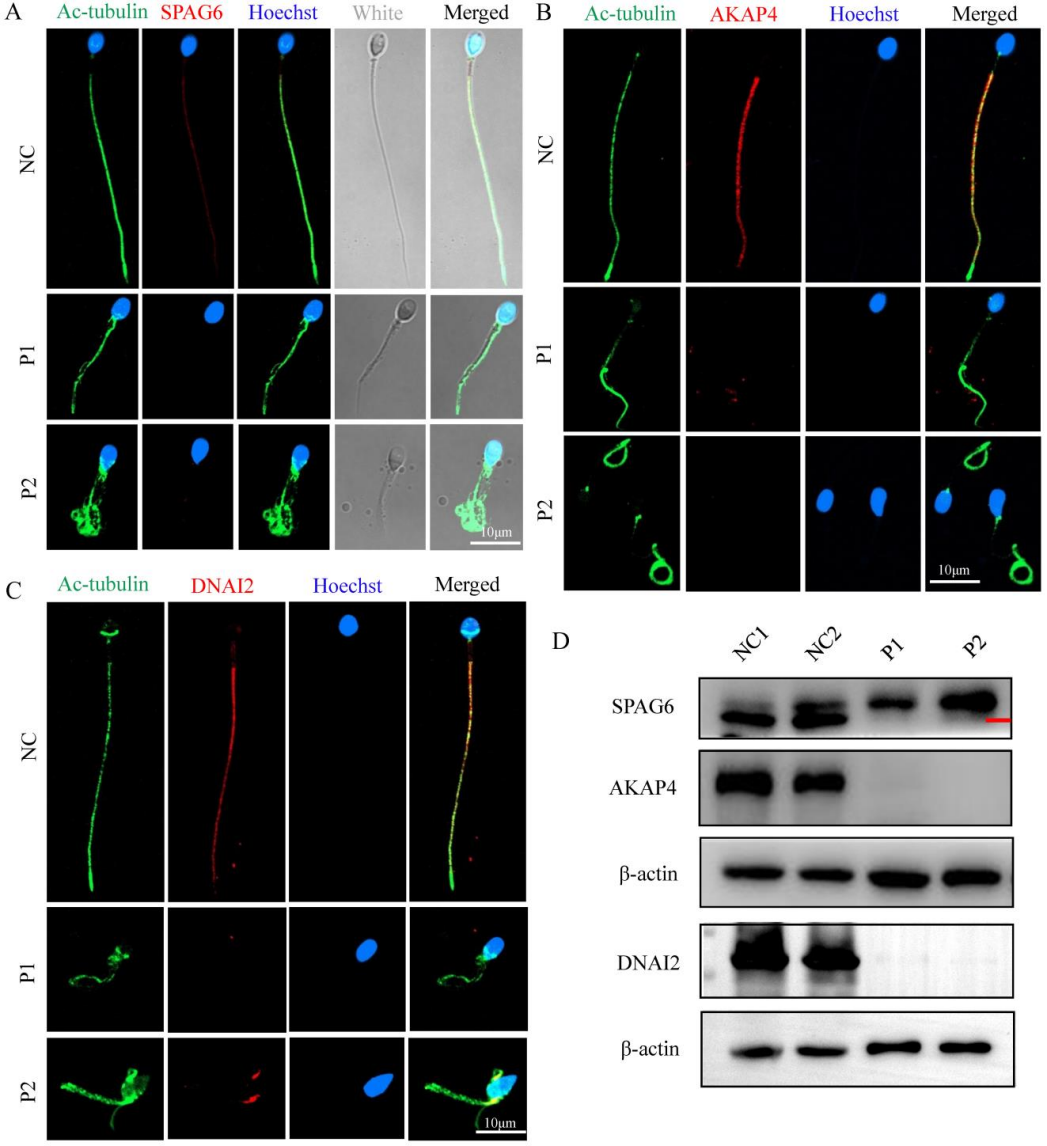
** The localization and levels of flagella associated proteins in* FSIP2* patients and controls. (A-C)** Immunofluorescence assays of flagella associated proteins including SPAG6 (a component of the CP complex), AKAP4 (a protein described to localize to the fibrous sheath), and DNAI2 (Dynein Axonemal Intermediate Chain 2) in *FSIP2* patients and controls. Anti-SPAG6 (red in A), anti- AKAP4 (red in B) and Anti- DNAI2 (red in C) normally localized along the sperm flagella in the control sperm. Signals of SPAG6, AKAP4 and DNAI2 were almost absent in sperm obtained from men harboring *FSIP2* variants. Anti-ac-tubulin (green) marked the sperm flagella. The nuclei of spermatozoa were Hoechst-labeled (blue). Scale bars: 10μm. **(D)** WB assays analysis the levels of SPAG6, AKAP4, and DNAI2 in sperm obtained from men harboring *FSIP2* variants and normal control. The results of WB assays were accordance with those of immunofluorescence assays described above.

To explore the ultrastructural defects observed in the principal piece, we measured the localizations and levels of different substructures of the FS and axoneme component proteins, including SPAG6 (a component of the CP complex), AKAP4 (a protein that localizes to the FS), and DNAI2 (dynein axonemal intermediate chain 2, a component of outer dynein arm (ODA)). We found that SPAG6, AKAP4, and DNAI2 signals were normally localized along the sperm flagella in control sperm but were almost absent in sperms of men carrying *FSIP2* variants (Fig. 7A, B, and D), which were confirmed by WB assays (Fig. 7D). These findings suggested that the CP component, FS, and outer dynein arm (ODA) are affected by variants in *FSIP2*.

**Figure 8:**
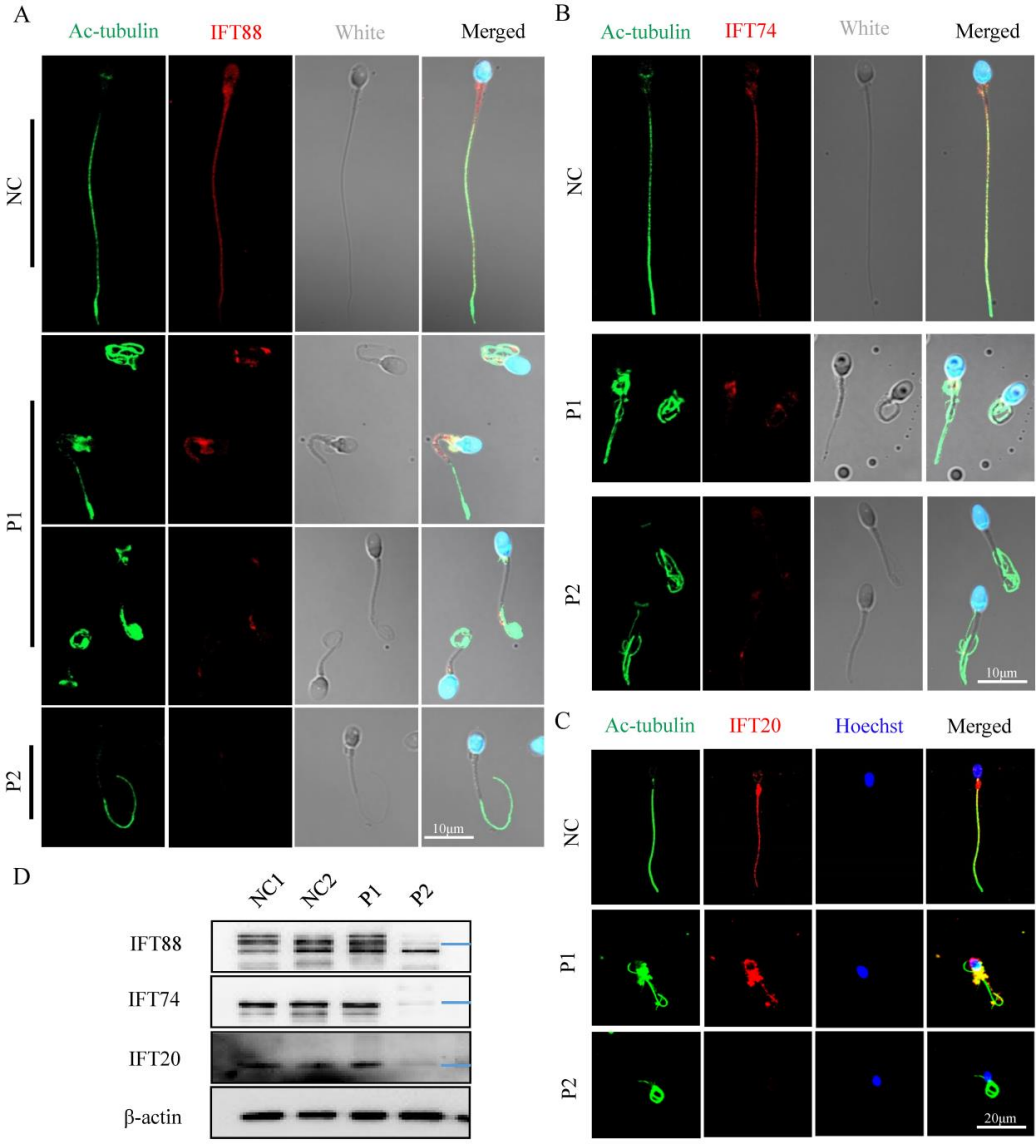
** The localization and levels of IFT-B related proteins (IFT88, IFT74, IFT20) staining signals in normal individual and *FSIP2*-deficient subjects. (A)** Immunofluorescence staining assays were performed on the spermatozoa using anti-IFT88 (red) and anti-α-tubulin (green) antibodies. DNA was counterstained with Hoechst as a nuclear marker. Scale bars: 10μm. **(B)** Immunofluorescence staining assays were performed on the spermatozoa using anti-IFT74 (red) and anti-α-tubulin (green) antibodies. DNA was counterstained with Hoechst as a nuclear marker. Scale bars: 10μm. **(C)** Immunofluorescence staining assays were performed on the spermatozoa using anti-IFT20 (red) and anti-α-tubulin (green) antibodies. DNA was counterstained with Hoechst as a nuclear marker. Scale bars: 20μm. **(D)** The levels of IFT88, IFT74 and IFT20 in sperm of normal individual and *FSIP2* deficient subjects were detected by WB assays, β-actin was used as internal reference.

The IFT is a highly conserved bidirectional cargo delivery system that transports cilia/flagella-associated proteins along axonemal MTs to assemble and maintain cilia/sperm flagella. IFT complexes consist of at least 22 highly conserved proteins that are divided into two subcomplexes: IFT-A (minus-end-directed transport) and IFT-B (plus-end-directed transport) 27. To determine the effect of *FSIP2* variants on the assembly of sperm flagellar axonemal protein complexes, the location and expression of the IFT-B subcomplex components IFT88, IFT74, and IFT20 were examined. IFT88, IFT74, and IFT20 staining signals were dislocated in P1 including concentrated at the mid-piece and distributed discontinuous along the flagella, and were almost absent in P2 (Fig. 8A, B and C). Western blots analysis in sperm lysates revealed that the levels of IFT88, IFT74 and IFT20 proteins in P1 were comparable with those in control and reduced significantly in P2 (Fig. 8D). These results may be related to the formation of truncated protein of FSIP2, which may affect the normal transport of IFTs. Collectively, our experimental observations indicate that FSIP2 is required for the normal assembly of the axoneme for the normal transport of flagella-associated proteins to the assembly site.

### Successful impregnation following intracytoplasmic sperm injection (ICSI) treatment of individuals carrying *FSIP2* variants

Three partners of men carrying *FSIP2* variants were unable to conceive spontaneously without contraception for more than one year. Majority of studies to date have demonstrated that ICSI treatment is an effective way of overcoming the physical limitations experienced by most individuals with asthenoteratozoospermia. The partner of study participant P1 had a normal basal endocrine assessment and a regular menstrual cycle. A total of 29 oocytes were retrieved following treatment with a gonadotropin-releasing hormone (GnRH) agonist. Of these, 24 metaphase II (MII) oocytes were microinjected, and 19 were successfully fertilized. Seven blastocysts were generated on the 5^th^ day and frozen for 6 months to prevent development of ovarian hyperstimulation syndrome. P1's partner became pregnant after the second frozen embryo transfer (Table 3).

Similarly, following treatment with an antagonist regimen, twelve oocytes were retrieved from the partner of study participant P2. Eleven MII oocytes were successfully microinjected, and five were fertilized and cleaved. Three high-quality blastocysts were subsequently frozen. Six months later, two blastocysts were transferred after thawing, resulting in pregnancy and detection of a single fetal heartbeat via ultrasound, and gave birth to a healthy child (Table 3). Unfortunately, information of the assisted reproductive cycle of the third couple was lost and the semen specimen could not be obtained for the molecular experiments above. Retrospect to the subject with the *FSIP2* variant (A011) we reported in 2018^32^, we found that the partner of A011 had a normal basal endocrine assessment and a regular menstrual cycle. Following treatment with an antagonist regimen, seventeen oocytes were retrieved and fourteen MII oocytes were successfully microinjected. Finally, eleven oocytes were fertilized and cleaved, and seven high-quality blastocysts were subsequently frozen. One blastocyst was transferred and a healthy baby was born. Overall, our study shows that male infertility caused by *FSIP2* variants can be rescued by ICSI treatment. Successful outcomes in these three couples provide guidance on clinical treatment options for patients with MMAF who carry *FSIP2* variations and similar variants and provide evidence of successfully applying assisted reproduction techniques to help patients with idiopathic asthenoteratozoospermia conceive.

## Discussion

In this study, we identified novel compound heterozygous *FSIP2* variants in three unrelated individuals in a cohort of 105 patients with asthenoteratozoospermia. FSIP2 has previously been associated with FS formation. Despite recent studies showing an association between the *FSIP2* truncated variant and the MMAF phenotype 31-33, 42, 53, many questions still remain. In this study, TEM analyses of sperm from individuals carrying *FSIP2* variants revealed severe dysplasia of FS, including missing FS and failure to form longitudinal FS columns (3^rd^ and 8^th^), leading to extremely disorganized and hypertrophic FS. These FS defects have previously been reported in patients carrying *FSIP2* and *AKAP4* variants and are termed DFS. These FS defects do not appear to be present in sperm cells of individuals with MMAF who have variants in the axonemal genes *DNAH1*, *SPAG6*, and *TTC21A* 12, 22, 54. Sperms of individuals carrying *FSIP2* variants also displayed some axonemal anomalies, including the absence of CP and the disorganization of DMT, and dynein arms, similar to those of individuals with MMAF who carry other gene variants. While the presence of multiple flagellar axonemes surrounding the mid-piece wrapped in excess residual cytoplasm had not been analyzed previously in subjects with MMAF harboring axonemal related gene variants. We also observed that staining signals associated with IFT-B-related proteins: IFT88, IFT74, and IFT20, were dislocated or absent in individuals with *FSIP2* deficiency. Protein-protein interaction network analysis by STRING showed that FSIP2 may be highly connected with FSIP1 and CFAP69. The protein level of IFT20 was downregulated significantly in the *Fsip1*^-/-^mice testis and FSIP1 interacted with IFT20 involving sperm flagellum assembly 42. Additionally, SPEF2 possible had the role in protein through the manchette towards the sperm tail interacting with IFT20 and CFAP69 55, 56. Thus, we can presume that FSIP2 is not only a structural protein of the FS but can also serve as an intra-flagellar transporter, binding to IFT20, one component of IFT-B complexes. This suggests that FSIP2 plays an important role in maintaining the structure and function of sperm flagellum.

In addition to the severe abnormalities of the axoneme and FS structures described above, sperm from patients carrying *FSIP2* variants also had elongated MS in the sperm mid-piece and the elevated levels of MS protein TOMM20. Mitochondrial ATP content also increased consistently, however, mitochondrial utilization decreased, suggesting that ATP producing systems were not affected. Previous study found that mitochondrial DNAs (mtDNAs) content were increased during meiosis and early spermatogenesis in the mouse testis and 8-10-fold of mtDNAs were eliminated at the time of differentiation of the round spermatids into elongated spermatids 35, 57, 58. *FSIP2* was transcribed in late spermatocyte development. We postulate that FSIP2 may associate with the process of mitochondria multiplication during meiosis and early spermatogenesis, or the process of the retention of mitochondria during differentiation of the round spermatids into elongated spermatids, little is known about it, which should be further study in the *Fsip2* KO mice. The reduced mitochondrial consumption may be resulted from the serious damaged flagellar structure of spermatozoa, inducing sperm more likely to immotile or to die after collection, and a partial decrease in mitochondrial membrane potential. The dynein, major ATP-consuming motor protein, may be disrupted due to *FSIP2* deficiency during sperm flagellar assembly, which may also probably responsible for the low ATP consumption.

Analysis of spermatogenesis in rats revealed that longitudinal FS columns begin to appear around the distal end of the axoneme immediately after axoneme initiation, and then extend 'backwards' as the axoneme extends. Following the movement of the annulus ring during spermatogenesis, the proximal region of ODFs and axoneme is exposed, allowing the mitochondria to assemble and generate the mid-piece, supplying energy that maintains sperm motility. Both mitochondrial length and gyre numbers are strictly regulated 27. However, the mechanisms underlying mitochondrial keeping or removing during spermatogenesis remain unclear. The annulus ring is located between the middle and principal pieces and is an important physical barrier to the intra-flagellar transport of spermatozoa 59-62. The annulus is often missing in sperm with primarily MS defects, not in MMAF. In this study, the lengthened MSs were thought to be the results of the missing annulus ring and the dislocation or absence of annulus-associated protein SEPT4 between the mid and principal pieces. However, we found that the lengthened MSs were not existing in sperm from individuals carrying *SEPT4* variants (Fig.S2B), indicating that the termination of MS extension may be determined by the FSIP2 protein, rather than the position of the annulus ring. This hypothesis was confirmed by that *Cfap157*-deficient sperm presented with axonemal loops, clustered mitochondria in the midpiece regions, abnormal distribution of fibrous sheath in the principle pieces and normal annulus rings between them 37. However, current data are far from definitive, and mechanism studies are required to further confirm the hypothesis.

Although knockout of *Cfap157*, *Armc12*, *Tbc1d21*, *Mff*, *Gk2*, *Sept4*, *Fsip1* and other genes in mouse had been reported to cause various malformation in sperm MS assembly, including mitochondrial aggregation, loose assembly, and short MS 37-41, 60. In humans, in addition to the specific flagellum defects presented in the MMAF phenotype, variations in *CFAP65* (MIM: 614270),* CFAP58* (MIM: 619129), *EIF4G1* (MIM: 600495), and *DNHD1* (MIM: 617277) also induced sperm MS abnormalities 19, 46-48. However, no variations had been reported to cause the lengthened MS sperm in human, the similar phenotype as in *FSIP2*.Different from *AKAP4*, *AKAP3*, and *FSIP1*, *FSIP2* was transcribed in late spermatocyte development 30. Deficiencies of *FSIP2* were associated with DFS, MMAF and globozoospermia 31-33, 42. Recurrent *FSIP2* amplification had been linked to testicular germ cell tumor 63, and *FSIP2* was also reported as a novel potential candidate gene for nonobstructive azoospermia 64. Here, we found that biallelic variants of *FSIP2* were not only associated with DFS, MMAF described previously, but also related to the multiple axoneme, the lengthened MS and lower mitochondrial ATP utilization. These findings indicated that *FSIP2* might play key roles during spermatogenesis. However, the mechanisms underlying *FSIP2* involving in mitochondrial keeping or removing, organization, and termination of MS extension during spermatogenesis remain unclear, which need to be further study.

In our study, the frameshift mutations M1 in P1, M3 and M4 in P2, M5 in P3 created a premature termination codon (PTC) downstream. The stop-gain mutation M2 in P2 also introduced a PTC at the place of mutation. 'Nonsense' and frameshift mutations are associated with low mRNA induced by nonsense-mediated mRNA decay (NMD) 65. The missense variant M6 (c.9043G>A (p.E3015K)) in P3 at the last part of exon 16 of *FSIP2* may affect the pre-mRNA splicing process lead to PTC and result in NMD 66, 67. Thus, the expression levels of *FSIP2* mRNA were reduced significantly in the three patients.

Previous reports identified seven homozygous and two compound heterozygous variants of *FSIP2* in individuals with MMAF. ICSI treatment outcomes in individuals carrying seven homozygous *FSIP2* variants were not included. The partner of one of the two patients carrying compound heterozygous *FSIP2* variants did not achieve pregnancy, whereas the other achieved good clinical outcomes after assisted reproductive therapy using ICSI. In this study, two of three men carrying compound heterozygous *FSIP2* variants and one subject harboring homozygous *FSIP2* variant we reported previously achieved successful ICSI outcomes (outcome information for the third patient was lost). Our findings can be used during genetic counseling of individuals with MMAF and asthenoteratozoospermia who carry *FSIP2* variants, prior to commencing ICSI treatment.

Although the findings of this study are interesting, the present study has some limitations, especially there was no* Fsip2* knockout (KO) mice model to detect the effect of *Fsip2* gene on the process of spermatogenesis. Therefore, identification of a larger number of individuals carrying *FSIP2* variants and generation of* Fsip2* KO mice are needed to elucidate the detail molecular mechanism of *Fsip2* gene influence on MS assembly, MS function and flagellum formation during spermatogenesis.

## Conclusion

Our experimental observations of humans carrying *FSIP2* variants indicate that *FSIP2* deficiency not only leads to severe dysplasia of the FS but also induces total destabilization of the axoneme, an MMAF phenotype caused by the axonemal gene. Extremely disorganized or missing FS led to a loss of the annulus and an elongated “super-length” MS. Moreover, successful pregnancy outcomes could be achieved following ICSI treatment of spermatozoa from men carrying *FSIP2* variants. Overall, our findings expand the variants spectrum of *FSIP2* and improve our understanding of the new phenotype of spermatozoa in men carrying *FSIP2* variants.

## Supplementary Material

Supplementary figures and tables.

## Figures and Tables

**Table 1:** Bi-allelic Variants of *FSIP2* Variants Identified in Chinese Men

*FSIP2* Variant	P1		P2		P3	
cDNA alteration	c.8104dup	c.4574C>A	c.8038dup	c.9234dup	c.10247dup	c.9043G>A
Variant allele	Het	Het	Het	Het	Het	Het
Protein alteration	p.L2702Pfs*44	p.S1525*	p.T2680Nfs*9	p.L3079Tfs*6	p.E3417Gfs*12	p.E3015K
Variant type	frameshift	stop-gain	frameshift	frameshift	frameshift	Missense
**Allele Frequency in Human Population**						
1000 Genomes						
gnomAD	NA^a^	NA^a^	9.1×10^-5^	NA^a^	7.0×10^-5^	NA^a^
gnomAD-EAS	NA^a^	NA^a^	0	NA^a^	0	NA^a^
**Function Prediction**						
SIFT	NA^b^	NA^b^	NA^b^	NA^b^	NA^b^	D
PolyPhen-2	NA^b^	NA^b^	NA^b^	NA^b^	NA^b^	NA^b^
Mutation Taster	NA^b^	NA^b^	NA^b^	NA^b^	NA^b^	D
CADD	NA^b^	35.0	NA^b^	NA^b^	NA^b^	22.3

The accession number of human *FSIP2* is GenBank: NM_173651.4. Full-length FSIP2 has 6907 amino acids. Abbreviations are as follows: Het, heterozygous; NA^a^, not available; NA^b^, not assessed; D, deleterious.

**Table 2:** Semen Characteristics and Sperm Morphology in Chinese Men with Bi-allelic *FSIP2* Mutations

Subject	P1	P2	P3	Reference Limits
Age	30	27	29	
**Semen Parameter**				
Semen volume (mL)	1.0	3.7	2.075	>1.5
Semen concentration (10^6^/mL)	47.08	63.1	57.325	>15.0
Motility (%)	5.99	25	13	>40.0
Progressive motility (%)	3.39	5.4	4.45	>32.0
**Sperm Morphology**				
Normal flagella (%)	0	0	/	>23.0
Absent flagella (%)	0.7	0	/	<5.0
Short flagella (%)	41.3	32.7	/	<1.0
Coiled flagella (%)	5.8	3.7	/	<17.0
Angulation (%)	2.4	1.5	/	<13.0
Thin (%)	50.0	62.1	/	
**Mid-piece Morphology**				
Insert (%)	0.5	1.5	/	
Angulation (%)	0.9	1.5	/	
Lengthened (%)	92.9	100		
ERC (%)	32.7	33.8		
**Head Morphology**				
Normal (%)	21.3	26.5	/	
Amorphous head (%)	71.3	66.2	/	
Vacuolated (%)	28.2	21.6	/	
Small acrosomes (%)	5.0	1.0	/	

**Table 3:** The clinical outcomes of *FSIP2* mutated subjects ICSI

	*FSIP2* Mutated Subjects			
No. of couples	4			
Subjects	P1	P2	P3	A011
Male age (year)	30	27	29	28
Female age (year)	29	27	28	27
No. of ICSI cycles	1	1	1	
No. of oocytes retrieved	29	12	/	19
No. of oocytes injected	24	11	/	14
Fertilization rate (%)	79.2 (19/24)	45.5(5/11)	/	78.6 (11/14)
Cleavage rate (%)	100(19/19)	100(5/5)	/	100 (11/11)
8-Cell formation rate (%)	63.2(12/19)	80(4/5)	/	72.7 (8/11)
Blastocyst formation rate (%)	36.8(7/19)	60(3/5)	/	72.7 (8/11)
High quality blastocyst rate (%)	15.8(3/19)	60(3/5)	/	63.6 (7/11)
No. of transfer cycles	2	1	/	1
Number of embryos transferred per cycle	2	1	/	1
Implantation rate (%)	25(1/4)	100(1/1)	/	100(1/1)
Clinical pregnancy rate per transfer cycle (%)	50(1/2)	100(1/1)	/	100(1/1)
Miscarriage rate (%)	50(1/2)	0	/	0
Live birth	Yes	Yes	/	Yes

## Abbreviations

MMAF: multiple morphological abnormalities of the sperm flagella
WES: whole-exome sequencing
H&E: Hematoxylin-Eosin
SEM: Scanning electron microscopy
TEM: transmission electron microscopy
RT-qPCR: quantitative real-time polymerase chain reaction
MS: mitochondrial sheath
FS: fibrous sheath
CP: central pair
DMT: doublets of microtubules
ODF: outer dense fibers
ERC: excess residual cytoplasm
IF: immunofluorescence
WB: western blot
