# Supplementary material for: Novel FSIP2 Variants Induce Super-Length Mitochondrial Sheath and Asthenoteratozoospermia in Humans
Source: Int J Biol Sci. 2023 Jan 1;19(2):393–411. doi: 10.7150/ijbs.76051 (PMC9830513; doi:10.7150/ijbs.76051)
Supplement: Supplementary file 1 — Supplementary figures and tables. [file ijbsv19p0393s1.pdf]

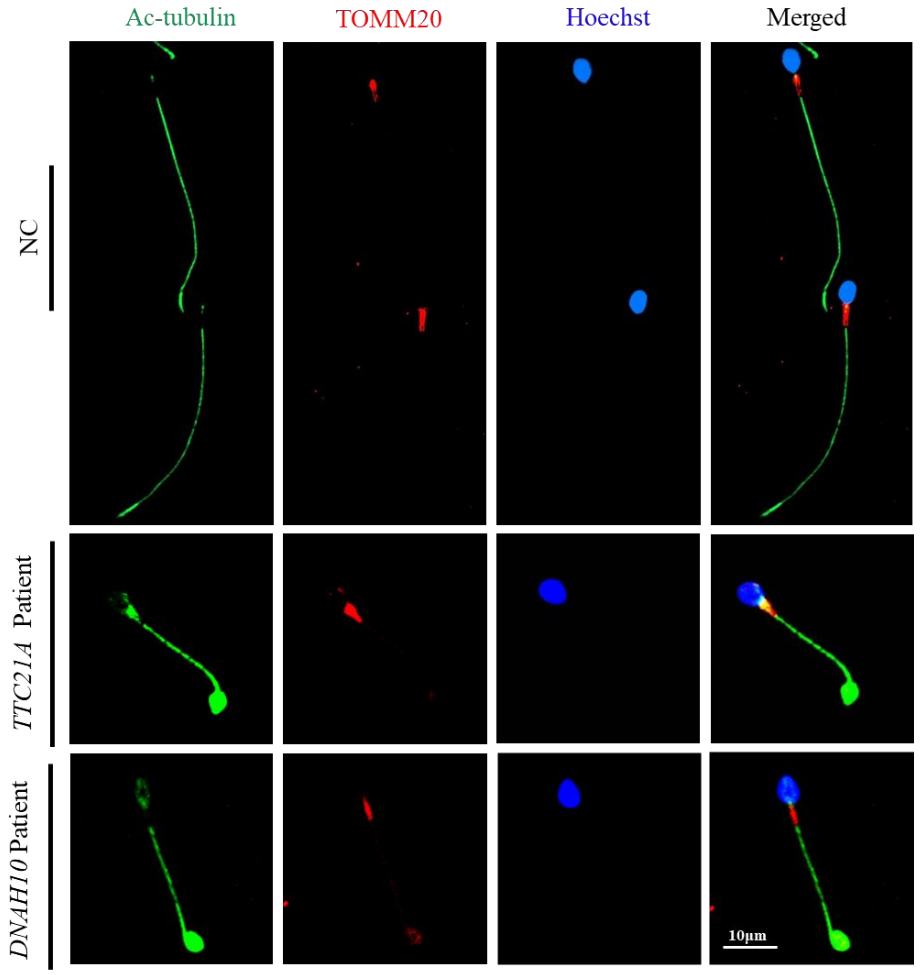

Figure S1. The immunofluorescence staining analysis of TOMM20 in sperm from subjects harboring *TTC21A* and *DNAH10*.Anti-TOMM20 (red) marked the sperm MS. Anti-ac-tubulin (green) marked the sperm flagella. The nuclei of spermatozoa were Hoechst-labeled (blue). Scale bars: 10 μm.

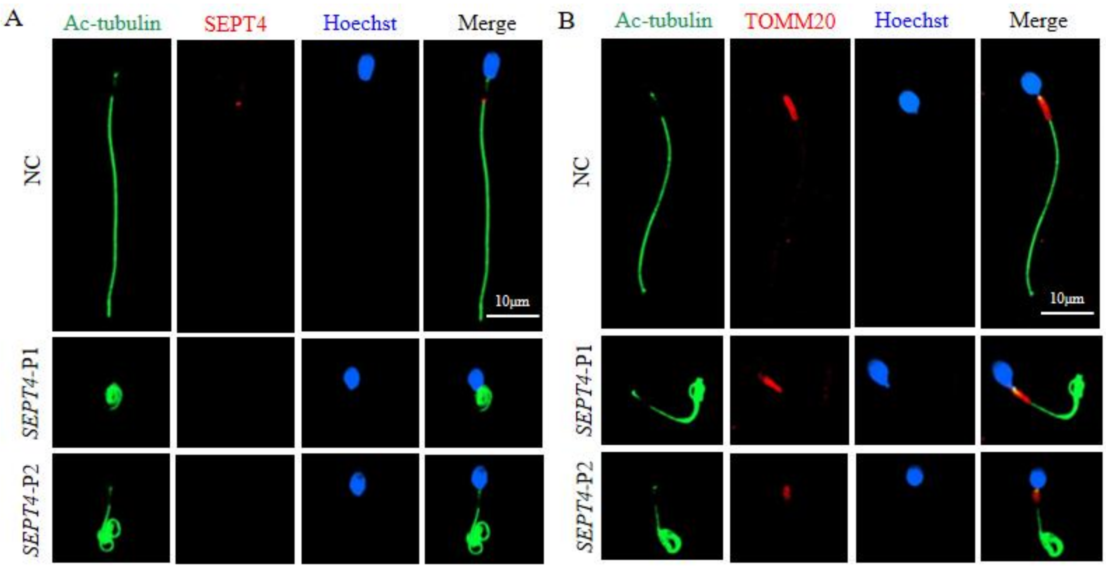

Figure S2. The localization and expression of annulus protein SEPT4 and the mitochondrial outer membrane protein TOMM20 in normal individual and *SEPT4*-deficient subjects.

(A) The presence and localization of SEPT4 staining signals in normal individual and *SEPT4*-deficient subjects. IF staining of the sperm from control subject showed strong SEPT4 signals at the sperm annulus, which located at the distal end of the mitochondria, the dot signals of SEPT4 were absent in *SEPT4*-deficient subjects. Anti-SEPT4 (red) marked sperm annulus ring. Anti-ac-tubulin (green) marked the sperm flagella. The nuclei of spermatozoa were Hoechst-labeled (blue). Scale bars: 10 μm.

(B) The immunofluorescence staining analysis of TOMM20 in *SEPT4* deficiency subjects. Anti-TOMM20 (red) marked the sperm MS. Anti-ac-tubulin (green) marked the sperm flagella. The nuclei of spermatozoa were Hoechst-labeled (blue). Scale bars: 10 μm.

Table S1. Primers Used for Amplification and Verification of *FSIP2* Mutations

Primer Names	Primer Sequences (5'-3')	Tm
M1-F	GTCTCACTTCCTTTAAAGGT	49℃
M1-R	CCTTCACAATTGTTTCTAGG	
M2-F	CAGCTATCTTGTAGTCAACA	52℃
M2-R	GAATATCTGAAGCTACAGCT	
M3-F	GCGATATCACAGGCTTATTCTTATGTCG	55℃
M3-R	AACCCAATGGCAGTCTTGGATCT	
M4-F	CATGTTAGAGTCATTTGTGGACTTGCAG	57℃
M4-R	TCCATTAGTGTGCCAATGATCTCAC	
M5-F	CTGGAGGGCAAAAGGATAACG	52℃
M5-R	GTGACATGTCTTGCTATCAGATGAACT	
M6-F	GCAACCAAATTGTTCAAGAGATTGTAG	56℃
M6-R	ACAGCAAGCATGTCACTGATGATATT	

Table S2. Primers Used for RT-qPCR Assays Primer

Primer Names	Primer Sequences (5'-3')	Tm
H- *FSIP2*-F	TCTCAGGAACAAAAGCCAGAGC	60℃
H- *FSIP2*-R	CTTGTTTGGAGACATCGGGC	
